# Response to imatinib as a function of target kinase expression in recurrent glioblastoma

**DOI:** 10.1186/2193-1801-3-111

**Published:** 2014-02-25

**Authors:** Marco Ronald Hassler, Mariam Vedadinejad, Birgit Flechl, Christine Haberler, Matthias Preusser, Johannes Andreas Hainfellner, Adelheid Wöhrer, Karin Ute Dieckmann, Karl Rössler, Richard Kast, Christine Marosi

**Affiliations:** Department of Internal Medicine I, Clinical Division of Oncology, 1-3 Comprehensive Cancer Center-Central Nervous System Tumors Unit (CCC-CNS), Medical University of Vienna, Vienna, Austria; Institute of Neurology, 1-3 Comprehensive Cancer Center-Central Nervous System Tumors Unit (CCC-CNS), Medical University of Vienna, Vienna, Austria; Department of Radiotherapy and Radiobiology, 1-3 Comprehensive Cancer Center-Central Nervous System Tumors Unit (CCC-CNS), Medical University of Vienna, Vienna, Austria; Department of Neurosurgery, District Hospital, Feldkirch, Austria; Department of Internal Medicine I, Clinical Division of Oncology, Währinger Gürtel 18-20, Vienna, 1090 Austria

**Keywords:** c-Abl, Arg kinase, c-Fms, c-kit, Cytokine, Glioblastoma, Inatinib, Markers, Platelet derived growth factor, Tyrosine kinase

## Abstract

**Background:**

Despite some progress in the treatment of glioblastoma, most patients experience tumor recurrence. Imatinib mesylate, a tyrosine kinase inhibitor of platelet derived growth factor receptor-alpha and -beta, c-fms, c-kit, abl and arg kinase (imatinib targets), has been shown to prevent tumor progression in early studies of recurrent gliomas, but has shown weak activity in randomized controlled trials. We studied the response to oral imatinib in 24 patients with recurrent glioblastoma who showed immunohistochemical expression of these imatinib targets in the initially resected tumor tissue.

**Methods:**

We offered oral imatinib, 400 mg once daily treatment to 24 recurrent glioblastoma patients whose initial biopsy showed presence of at least one imatinib inhibitable tyrosine kinase.

**Results:**

Six imatinib treated patients survived over one year. Twelve patients achieved at least tumor stabilisations from 2.6 months to 13.4 months. Median progression free survival was 3 months and median overall survival was 6 months. Imatinib was well tolerated. We found evidence, though not statistically significant, that arg kinase [Abl-2] immunopositivity had shorter survival [5 months] than the arg kinase immunonegative group [9 months].

**Conclusions:**

Responses to imatinib observed in this patient series where imatinib inhibitable tyrosine kinases were documented on the original biopsy are marginally better than that previously reported in imatinib treatment of unselected recurrent glioblastoma patients. We thus present a suggestion for defining a patient sub-population who might potentially benefit from imatinib.

## Introduction

Over the last ten years overall survival (OS) in glioblastoma (GB) after initial surgery has improved somewhat. After diagnosis of GB standard therapy consists in maximal feasible resection, followed by radiotherapy and concomitant adjuvant therapy with temozolomide (TMZ) (Stupp et al. 2009; Minniti et al. 2008; Taphoorn and Bottomley 2005), applicable also in older patients (Minniti et al. 2008) albeit resulting in shorter OS than in younger cohorts. This approach has led to an improved overall survival (OS) rate from 11 months to 15 to 20 months currently (Stupp et al. 2009; Minniti et al. 2008; Taphoorn and Bottomley 2005; Wöhrer et al. 2009). Clearly more is needed.

Almost all patients develop recurrences within two years after diagnosis. There is no current standard or established treatment for recurrent GB. Increasingly we are seeing patients with recurrent GB that are in better clinical condition than we saw ten years ago and who wish for and could tolerate additions to Treatment Options.

At recurrence, for each patient there are important individual treatment decisions depending on: i) clinical condition, ii) localisation of recurrence that determines suitability for second resection, iii) time elapsed since initial treatment to determine potential usefulness of re-irradiation, iv) methylation status of the methyl guanine methyl transferase (MGMT) gene promoter, v) potential for inclusion of the patient in an investigational treatment trial, and perhaps most importantly, vi) patient preference for the various risk-benefit options.

One of the first tyrosine kinase (TK) inhibitors tested in recurrent GB was imatinib (Gleevec® or Glivec®) (Wen et al. 2006). For an excellent review of imatinib’s development, kinase inhibition attributes, mechanism of action, and early clinical results, see ref. (Waller 2010). Imatinib inhibits several important TK’s that have been shown to be active in promoting GB growth such as platelet derived growth factor receptor (PDGFR) -α and -β (George 2003), c-Abl kinase (Panjarian et al. 2013), c-kit (Lennartsson and Ronnstrand 2012), arg (Mader et al. 2011; Beaty et al. 2013), and c-Fms, (Mouchemore and Pixley 2012). c-Abl kinase is a non-receptor TK where cytoplasmic activity is cell survival promoting yet nuclear activity is cell death promoting (Panjarian et al. 2013). c-kit, synonymous with CD117, is an outer cell-surface tyrosine kinase transmembrane receptor for 18 kDa stem cell factor (Lennartsson and Ronnstrand 2012), and c-fms is a cell surface receptor for 108 kDa colony stimulating factor-1, also known as macrophage-colony stimulating factor (Mouchemore and Pixley 2012). arg is the Abelson-related gene product, (same as Abl-related nonreceptor tyrosine kinase Arg, or Abl2), a large nonreceptor TK (Mader et al. 2011; Beaty et al. 2013). Of particular note arg, although commonly seen to be an element promoting cancer cell invasion [as in breast cancer 10, 11], can in some cancers work to arrest invasion (Hayes et al. 2012). We outline below results that could indicate GB might be another such cancer, complicating the use of imatinib.

Focal expression of PDGFR-alpha protein occurs in 25% of unselected GB’s, PGFR-beta in 19%, c-kit in 4%, and c-abl in 7% (Haberler et al. 2006). c-fms is expressed in glioblastoma but to what degree or frequency hasn’t been determined (Alterman and Stanley 1994).

Initial enthusiasm for imatinib was based on robust pre-clinical evidence (Wen et al. 2006; Waller 2010; Morris and Abrey 2010) but subsequent weakness of imatinib in clinical trials in unselected recurrent GB has since dampened enthusiasm. In single agent studies, both using 400 mg p.o. twice daily, Raymond et al. found imatinib gave a 16% progression free survival at six months (Raymond et al. 2008) while Wen et al. found a 10% progression free survival at six months (Wen et al. 2006). Yet two independent studies documented good GB tissue levels of imatinib and its primary active metabolite, approximately equal to or in some cases greater than blood levels (Razis et al. 2009; Holdhoff et al. 2010). This high tumor tissue imatinib level was concordant with previous murine studies (Tan et al. 2011; Soo et al. 2010).

Histologically glioblastoma has been traditionally diagnosed by presence of nuclear atypia, focal necrosis, florid microvascular proliferation, and frequent mitotic figures. Examination of mRNA expression patterns now allows division of GB into molecular genetic subtypes, 1) proneural, 2) neural, 3) classic, and 4) mesenchymal (Dunn et al. 2012; Verhaak et al. 2010). The proneural subtype consists of glioblastomas harbouring *TP53* mutations occurring mostly in younger patients and commonly found together with isocitrate dehydrogenase (IDH) mutation and PDFGR-alpha overexpression (Dunn et al. 2012; Verhaak et al. 2010). We speculated that proneural subtype would preferentially benefit from imatinib by virtue of having relatively higher dependence on dysregulated imatinib targets. As the percentage of proneural GBM is in the range of 12%, this could explain why the percentage of patients responding to imatinib in unselected series is remains low.

Based on in vitro data and on favourable clinical experience gained on Viennese patients participating in the EORTC study 16011 [clinicaltrials.gov] and some additional patients with advanced brain tumors treated with imatinib on a compassionate use basis, we offered imatinib to recurrent GB patients who were no longer candidates for alkylating therapies and who had positive immunohistochemical staining of PDGFR-α, or -β, or c-Abl, or c-kit, or c-fms. We report here on these patients with recurrent GB treated with imatinib.

## Patients and methods

### Patient eligibility

Entry requirements were recurrent GB, recurrent during or shortly after treatment with alkylating agents equal or less than three months after initial treatment ended and who had tissue available for immunohistochemistry. Of note, the analysis of the promoter methylation of the gene methylguanine-methytransferase (MGMT) was not done at our centre. Imatinib was offered only when primary resection tissue was positive on immunohistochemistry for one or more of the imatininb targets- PDGF-R α or -β, c-abl, c-kit, arg, c-fms.

GB recurrence had to be diagnosed on recent contrast enhanced magnetic resonance imaging scan (MRI). Patients were required to have no neurosurgical and or radiotherapeutic option. They had to be aged 18 years or older with a performance status ≤ 2 WHO score. Patients needed to have recovered from all toxicities from previous therapies, to present with stable or decreasing doses of corticosteroids for at least one week before start of therapy and to have adequate bone marrow, hepatic and renal function (leukocyte count > 3,000/μL and a platelet count > 100,000/μL; ALAT, ASAT, and alkaline phosphatase levels < two times upper limit of normal; bilirubin, blood urea nitrogen and creatinine levels < 1.5 times of institutional normal levels).

### Study design

This was an open label single centre named patient study. There was no limit on the number of prior therapies or number of previous tumor progressions. The primary endpoint was survival duration after treatment start with imatinib (OS), secondary endpoints were progression free survival (PFS) and the rate of PFS after 6 months of imatinib (PFS-6) and safety. The protocol was reviewed and approved by the IRB of the Medical University of Vienna, Austria.

### Treatment intervention

Imatinib was given at 400 mg fixed dose per day on a continuous oral dosing schedule until tumor progression, unacceptable toxicity, or consent withdrawal occurred. We did not have the opportunity to enhance the dosage of imatinib in patients under enzyme inducing antiepileptic drugs (EIAEDs), as there was no reimbursement for increased doses. After the third patient, all further patients were also given the proton pump inhibitor pantoprazol, 40 mg. in the morning, in order to minimize gastro-intestinal side effects.

### Treatment evaluation

Toxicity was evaluated according to the National Cancer Institute (NCI) common toxicity criteria (CTC) 4.0 (Franklin et al. 1994; Trotti et al. 2003) during routine monthly meetings, or at any time point when clinically indicated. Safety assessments including monitoring of serum chemistry and blood cell counts were done in bi-weekly intervals at therapy start, extended to monthly intervals after the first month.

Patients were monitored for treatment response with clinical evaluation at monthly intervals and every three months with contrast enhanced MRI scans. Response evaluation was based on MacDonald’s criteria (Macdonald et al. 1990).

### Immunohistochemistry

Immunohistochemical expression of PDGFR-α, -β, c-kit, c-abl and arg was determined in paraffin-embedded tumor specimens, fixed in 4% buffered formalin, as described previously (Haberler et al. 2006). The following antibodies were used at the indicated dilutions: polyclonal rabbit anti-PDGFR-α antibody (sc-338, Santa Cruz Biotechnology, Inc; 1:500), polyclonal rabbit anti-PDGFR-β antibody (sc-339, Santa Cruz Biotechnology, Inc; 1:500), polyclonal rabbit anti-human c-kit antibody (A4502, Dako, Glostrup Denmark; 1:400), polyclonal rabbit anti-c-abl antibody (sc-887, Santa Cruz Biotechnology, Inc; 1:1000) and polyclonal goat anti-arg antibody (sc-6356, Santa Cruz Biotechnology, Inc; 1:50). Additionally, phosphorylated epitopes of PDGFR-α, -β, c-kit and abl were analyzed using a polyclonal rabbit anti-PDGFR-α antibody (sc-12910, Santa Cruz Biotechnology, Inc; 1.50), a monoclonal mouse anti- PDGFR-β antibody (#3166, Cell Signalling Technology, Inc; 1:20) a polyclonal rabbit anti-c-kit antibody (#3991, Cell Signalling Technology, Inc; 1:25), and a polyclonal rabbit anti-c-abl antibody (#2864, Cell Signalling Technology, Inc; 1:250).

Assessment of PDGFR-α, -β, c-kit, c-abl and arg expression pattern was done semi-quantitatively and scored as widespread (>50%), moderate (50–10%), scant (<10%), or negative labelling of tumor cells. Only tumor cells with an intense cell-membrane-bound and/or intracytoplasmic immunoreactivity were evaluated as positive. A very faint, smudgy or nuclear staining was not considered as positive.

### IDH1 mutation

Formalin-fixed and paraffin-embedded tumor tissue blocks were cut at a thickness of 3-4 microns. Sections underwent heat-induced antigen retrieval for 60 minutes and incubated with the monoclonal IDH1-R132H antibody (clone DIA-H09, Dianova, Hamburg, Germany) at a dilution of 1:30 for 60 minutes. Detection of immunolabelling was performed using the Flex + Mouse system (Dako, Glostrup, Denmark) with diaminobenzidin as chromogen. Presence or absence of tumor cell immunolabelling was evaluated by one observer (A.W.). No case with partly positive and partly negative staining of tumor cells was encountered.

### Statistical considerations

The primary objective was to evaluate duration of survival of patients whose tumors had a positive staining with “imatinib targets” at the initial diagnosis of glioma from the day of starting imatinib 400 mg per day to the day of death (OS), further, the duration of diagnosis of tumor progression by imaging or the first day of clinical deterioration associated with tumor progression or unexplained death for any cause (PFS). Furthermore, the rate of 6-month progression free survival and the duration of overall survival were calculated using the Kaplan Meier method.

## Results

### Patient characteristics

Twenty-four patients fulfilling eligibility criteria were treated with imatinib. Average age was 53 years, with male to female ratio of 13:11. Eleven patients received imatinib as 2^nd^; nine patients as 3^rd^ line therapy; two patients as 4^th^ and two patients as 5^th^ line therapy. Patients’ characteristics are summarized in Table 1.

**Table 1:** **Patient characteristics**

Primary GBM	24 (100%)
Sex – n (%)	
Female	11 (45%)
Male	13 (55%)
Age – yr	
Median (Range)	53 (18 – 72y)
Performance score – n (%)	
WHO 0	0
WHO I	16 (65%)
WHO II	8 (33%)
Extent of surgery – n (%)	
Biopsy	3 (12.5%)
Partial resection	11 (46%)
Gross total resection	10 (41.5%)
Previous chemotherapies	
1	11 (46%)
2	9 (36%)
3	2 (3.5%)
4	2 (3.5%)
Antiepileptic drugs	
None	10 (41.5%)
EIAEDs	10 (41.5%)
Non-EIAEDs	4 (18%)

GBM - glioblastoma multiforme.

EIAEDs - enzyme inducing antiepileptic drugs.

n - Number of patients.

Patients started imatinib at a median 10.5 months after the first diagnosis, and after treatment with at least one alkylating compound. The median duration of therapy with imatinib was 3 months. Six of the 24 patients survived, and were treated for a year or more.

### Toxicity

Side effects of imatinib therapy consisted in transient peripheral oedema of legs or eye lids in six patients (25%), mainly occurring shortly after start of therapy or concomitantly to minor infections, i.e. of urinary tract. After complaints of three patients about abdominal pain, we started prescribing proton pump inhibitors to all patients and these effects were not reported any longer. Nausea and vertigo were reported by one patient each. No side effects exceed CTC grade 2 and were mainly of transient character. No patient had to stop imatinib because of toxicity and none withdrew consent.

### Immunohistochemistry

Table 2 lists the frequencies of expression of imatinib targets according to our semiquantitative scoring system. Tumor tissue for all the mentioned immunohistochemical analysis was available in 23/24 patients. In a patient with a small biopsy only, not all analyses could be performed. None of the 24 recurrent GB patients expressed c-kit. IDH1 mutation was tested in 19/24 patients and positive in only three.

**Table 2:** **Immunohistochemical markers and response to imatinib therapy**

Pat. no.	Sex	Age (y)	Delay to Th start (m)	Th duration (m)	Best response	PFS m	Survival m	Arg	pabl	abl	Pc-kit	c-kit	pPDGFR-β	PDGFR-β	PDGFR-α	pPDGFR-α	IDH-Ak
1	m	18,2	6,8	3	PD	3	6,2	neg	<10%	<10%	<10%	neg	neg	neg	10–50%	<10%	+
2	m	27,4	6,4	2,5	PD	2,5	2,5	neg	neg	neg	neg	neg	neg	neg	<10%	<10%	-
3	f	59,4	4,5	1,6	PD	1.6	3,2	<10%	neg	neg	neg	neg	neg	neg	<10%	<10%	-
4	m	48,5	5,4	1,7	PD	1,7	1,7	<10%	neg	<10%	neg	neg	neg	neg	neg	<10%	-
5	f	49,8	13	1	PD	1	1	neg	neg	neg	neg	neg	neg	neg	<10%	n.a.	-
6	m	60,9	25,1	0,6	PD	0.6	1,7	<10%	10–50%	10–50%	<10%	neg	<10%	neg	<10%	<10%	+
7	m	50,5	15,6	0,8	PD	0,8	2,1	neg	neg	neg	<10%	neg	neg	neg	neg	Neg	-
8	m	63,4	7,5	2	PD	2	11,7	<10%	neg	neg	<10%	neg	<10%	<10%	<10%	<10%	na
9	m	56,1	4,7	1,5	PD	1,4	3,3	<10%	neg	<10%	neg	neg	neg	neg	<10%	<10%	-
10	m	56,1	17,6	0,8	PD	0,8	0,8	<10%	neg	neg	10–50%	neg	>50%	neg	>50%	>50%	-
11	f	71,6	1	1,8	PD	1,8	1,8	neg	neg	neg	<10%	neg	neg	neg	10–50%	10–50%	na
12	f	39	24,7	1,1	PD	1,1	1,1	<10%	<10%	<10%	neg	neg	neg	neg	<10%	<10%	-
13	m	62,6	20,8	0,9	SD > 6m	7,9	17,1	neg	neg	neg	<10%	neg	<10%	neg	neg	<10%	-
14	f	41	8,5	5,9	SD < 6m	5,9	10,4	neg	neg	neg	10–50%	neg	neg	neg	<10%	10–50%	-
15	f	42,2	20	2,2	SD < 6m	2,6	4,6	neg	neg	neg	neg	neg	neg	neg	<10%	<10%	-
16	m	56,9	4,3	5,8	SD < 6m	5,8	16,6	neg	neg	neg	neg	neg	neg	neg	<10%	10–50%	-
17	m	70,2	4,3	5,6	SD > 6m	5.6	13,4	neg	10–50%	10–50%	<10%	neg	neg	neg	<10%	>50%	-
18	f	67	19,6	13,1	SD > 6m	13,1	13,1	10–50%	neg	neg	<10%	neg	<10%	neg	10–50%	>50%	na
19	f	62	4,3	8,1	SD > 6m	8,1	8,1	<10%	10–50%	neg	neg	neg	neg	neg	neg	Neg	na
20	f	48,6	4,4	6,2	SD > 6m	6,2	6,2	n.a.	n.a.	n.a.	n.a.	neg.	<10%	n.a.	<10%	n.a.	na
21	m	59,5	8,8	8,9	SD > 6m	8,9	8,9	neg	neg	neg	neg	neg	neg	neg	<10%	Neg	-
22	m	68,4	12,2	9,4	SD > 6m	9,4	9,4	neg	<10%	<10%	neg	neg	neg	neg	<10%	<10%	-
23	f	32,1	14,5	7,3	PR	60	60	neg	neg	neg	neg	neg	neg	neg	<10%	<10%	+
24	f	61,6	35,7	14,4	PR	14,4	32	neg	neg	neg	neg	neg	<10%	neg	<10%	Neg	-

GBM - glioblastoma multiforme.

PD - progressive disease.

SD - stable disease < and > 6 months.

PR - partial response.

PFS - progression free survival after start of imatinib.

Survival: duration in months after start of imatinib.

m: months.

p: antibody against the phosphorylated form of a tyrosine kinase.

“<10%”: fewer than 10% of tumor cells expressed marker.

“neg”: assay was done and no reactive cells were found.

n.e.: not evaluable for response.

pt. 15: therapy stopped due to toxicities: ooedema, therapy stopped after 4 weeks.

### Treatment outcome

Two patients achieved PR; one of them (nr.23 Table 2) was a women aged 61 years at initial diagnosis of glioblastoma with 4 cm diameter in the right frontal lobe that underwent a partial resection and later standard alkylating therapy (at this time with CCNU for 8 cycles of 42 days) and survived without progression until 35 months after initial diagnosis. In addition, her tumor showed an IDH1 mutation.

The other patient, a young, female patient aged 32y (nr 24 Table 2) was diagnosed with a more than 5 cm in diameter left frontal GBM, underwent biopsy only followed by concomitant and adjuvant therapy with Fotemustine/Dacarbacine (8 cycles). Three months later her MRI scan showed an increasing contrast enhancement (7 months after Initial diagnosis) and she received one cycle of Temozolomide 150 mg days 1–5 and because of severe pancytopenia and expression of imatinib targets was then given Imatinib. By retrospect, the increasing contrast enhancing mass could have been pseudoprogression; but it remains exceptional that she survived without any progression for more than 60 months after a single adjuvant cycle of temozolomide.

**Figure 1:**
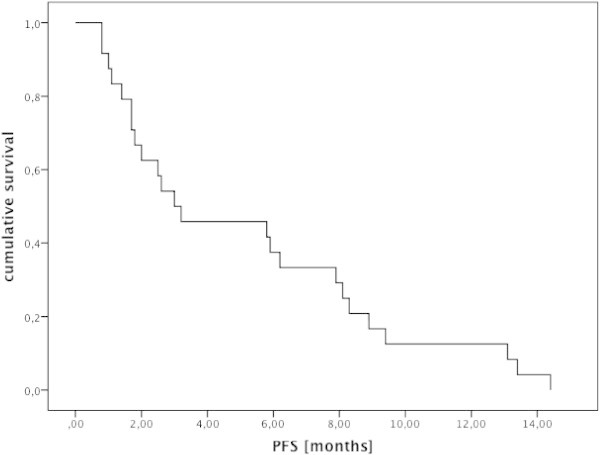
**Kaplan Meier plot showing duration of progressive free survival from the start of imatinib to progression of GBM in 24 patients.**

**Figure 2:**
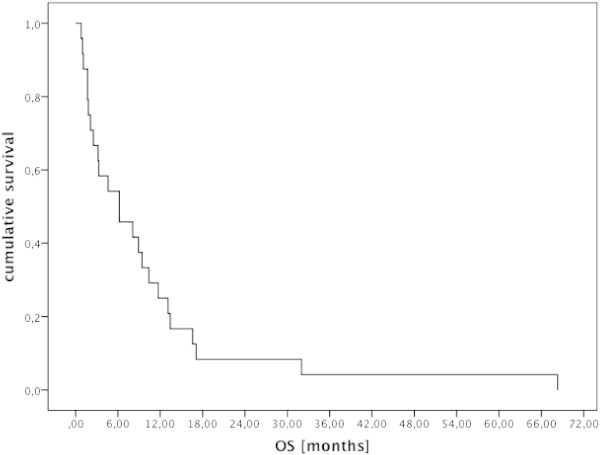
**Kaplan Meier plot showing overall survival in patients with GBM from start of imatinib.**

**Figure 3:**
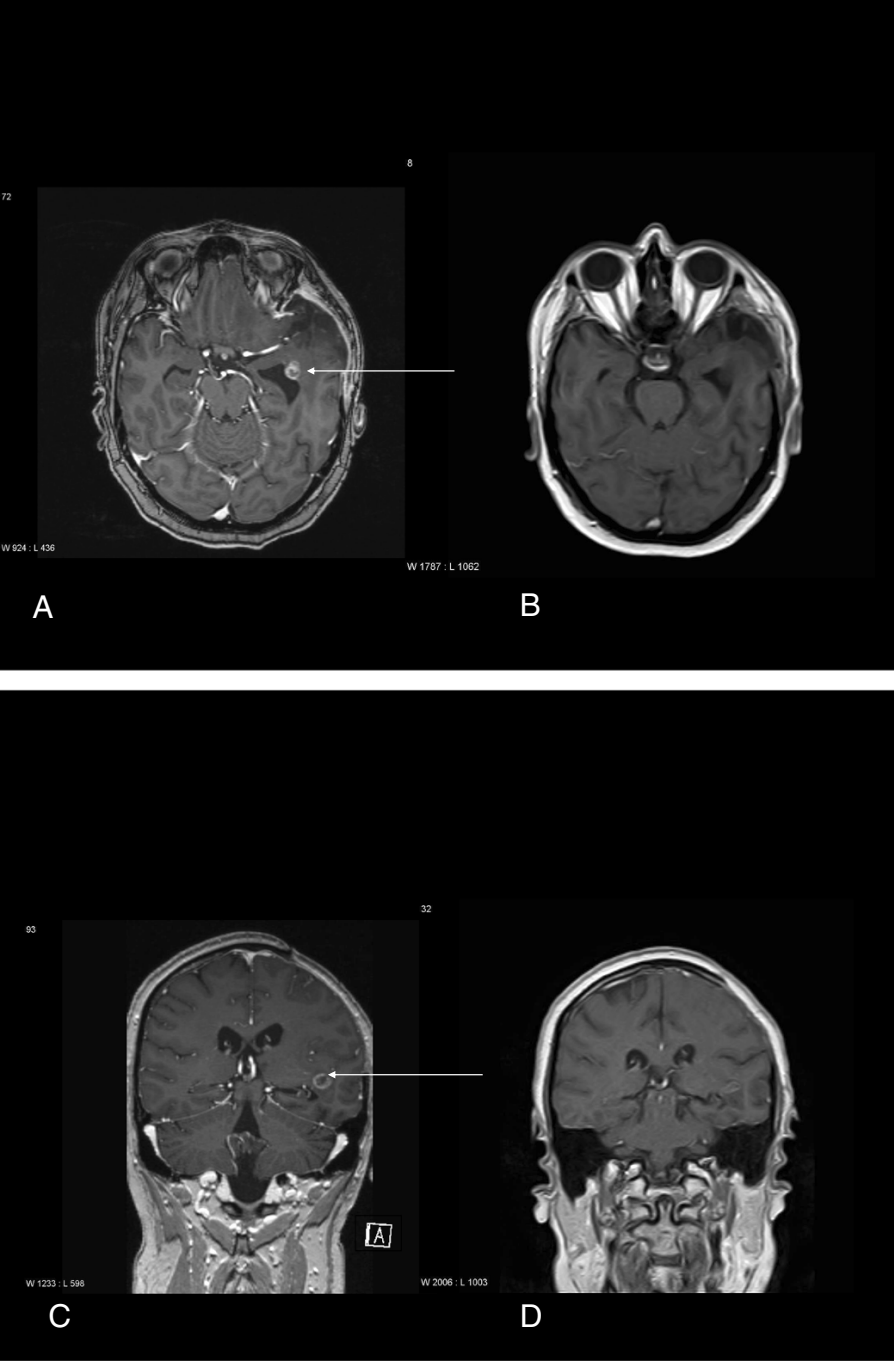
**MRI slides of patient with major response.** T1 weighted, contrast enhanced MRT. **A**: Horizontal: before start of imatinib: with a left frontal lesion with contrast enhancement. **B**: Horizontal: 3 months after start of imatinib, contrast enhancement of the lesion is not longer visible. **C**: coronal, before start of imatinib with the contrast enhancing lesion near the ventricle. **D**: coronal, 3 months after start of imatinib: no contrast enhancing lesion visible. The best fitted sections were selected for this image, as the head positioning and bending of the neck were not exactly similar in both examinations.

Ten additional patients reached stable disease, seven for more than 6 months and up to thirteen months. Twelve patients (50%) showed progressive disease at the first scan. The median overall survival after the start of imatinib was 6.2 months and the median duration of PFS was three months (see Figures 1 and 2). Patients responding to imatinib showed rapid clinical improvement with subjective relief of symptoms within two weeks and objective regression of contrast enhancing lesions in MRI, as shown for one of the patients with major response (Figure 3).

We found no correlation between numbers of imatinib targets positive, or with the percentage of immunohistochemical staining cells for a given target and OS. Average number of targets positive, 3, was the same for the 11 patients with OS <6 months as the 13 patients with OS > 6 months. We found no particular pattern of imatinib targets positive or negative that predicted longer OS other than arg, where negative staining predicted a slightly longer OS (9 months in 14 patients) than immunopositive patients (5 months in 9 patients).

## Discussion

In this series we treated 24 glioblastoma patients with early recurrence that had immunohistochemical expression of imatinib targets in the initial tumor biopsy. We observed marginally better results than that reported in previous series of monotherapy with imatinib, Raymond et al. (giving 400 mg twice daily) saw 5.2 months OS compared to our 6.2 (Raymond et al. 2008). Wen et al. saw 5.2 month overall average, 3.1 month median PFS, 3% (1/33 patients) progression free at 6 months compared to our 33% (8/24) (Wen et al. 2006).

We saw 6/24 patients surviving more than one year but were unable to identify any global TK pattern that differentiated them from the 7/24 surviving less than two months. However we did see a potentially interesting OS of 9 months in patients whose biopsy immunostained negative for the kinase arg (see Table 2) compared to OS of 5 months for those staining positive, but this did not reach significance. Furthermore within this study we were not able to assess whether the chosen targets were overexpressed or amplified, nor their potential action on downstream targets.

However, we consider the possibility that GB is one of the cancers where arg kinase activity inhibits invadopodia activity similarly as found in head and neck cancers (Hayes et al. 2012) and therefore whose inhibition would be undesirable. A follow up study will exclude patients with positive arg biopsies from imatinib treatment in case this association is not ephemeral. Our numbers were too small to statistically exclude or confirm such association.

The toxicity related to imatinib intake was generally low. Of note, neither clinically significant cytopenias, hepatic toxicity, nor cardiotoxicity were observed. This might be due to the fact that most of the glioma patients are using imatinib for shorter periods than would be typical in patients with chronic myeloid leukemia. We also saw less cytopenias than previous imatinib studies, perhaps due to our use of 400 mg per day as opposed to 400 mg twice daily in previous studies (Wen et al. 2006; Raymond et al. 2008). In this regard the potential for hormesis (Cox 2006; Calabrese 2012) must be considered. Although we intuitively think of “more drug = greater effect” this does not always hold. Hormesis- the U shaped [or inverted U shaped curve] dose–response curve is not rare where increasing dose after a given point can decrease total cytotoxicity, as with ciprofloxacin in vitro (Hincal et al. 2003). Thus the lower dose we used compared to the previous studies of (Wen et al. 2006) and (Raymond et al. 2008) could account for better effect were hormesis to be active in this dose range.

Randomized prospective trials are needed to confirm this finding. Even if such trials confirm the small benefit we saw, clearly augmentation strategies will be needed for imatinib. To this end Soo et al. demonstrated three fold increase in brain imatinib levels if co-administered with the anti-malaria drug primaquine (Soo et al. 2010), Tan et al. found 3.9 times the brain imatinib levels in mice co-administered the anti-anaerobic antibiotic metronidazole (Tan et al. 2011). Since both metronidazole and primaquine are well-tolerated drugs with which we have decades of experience, these might be easy ways to augment imatinib’s therapeutic index in treating GB. On the other hand if hormesis is demonstrated such increased brain tissue levels could be counterproductive. Only further research can resolve this matter.

A facinating study by Coniglio et al. showed that c-fms, an imatinib inhibitable TK, secreted by GB cells strongly enhanced otherwise normal brain microglia’s infiltration into the growing tumor as well as the concomitant centrifugal counter-migration of glioblastoma cells (Coniglio et al. 2012). Epidermal growth factor receptor (EGFR, also termed HER-1) stimulation enhanced glioblastoma cells’ countermigration into surrounding brain [34], suggesting that the EGFR inhibitor erlotinib may be synergistic with imatinib in suppressing glioblastoma growth.

The high degree of spatial localization of over-expression of PDGFRs makes sampling error risk high (Szerlip et al. 2012). The larger the tumor tissue we have to examine the more reliable will be the determination of TK expression pattern. In confirming the tremendous heterogeneity within an individual glioblastoma, Little et al. found to some degree that areas of EGFR and areas of PDGFR overexpression tended to be mutually exclusive in human GB biopsy tissue (Little et al. 2012), again indicating potential for erlotinib to increase imatinib’s effectiveness.

## Conclusion

We showed marginal benefit of imatinib treatment of recurrent glioblastomas expressing imatinib inhibitable TKs. Our results were somewhat better than that found in previous studies of unselected patients. We offer several paths that might enhance imatinib effectiveness.

## Abbreviations

BBB: Blood brain barrier
MGMT: Methyl guanine methyl transferase
OS: Overall survival
PDGFR: Platelet derived growth factor receptor
PFS: Progression free survival
QOL: Quality of life
TK: Tyrosine kinase.

## References

[CR1] Alterman RL, Stanley ER (1994). Colony stimulating factor-1 expression in human glioma. Mol Chem Neuropathol.

[CR2] Beaty BT, Sharma VP, Bravo-Cordero JJ, Simpson MA, Eddy RJ, Koleske AJ, Condeelis J (2013). β1 integrin regulates Arg to promote invadopodial maturation and matrix degradation. Mol Biol Cell.

[CR3] Calabrese EJ (2012). Hormesis and the salk polio vaccine. Dose Response.

[CR4] Coniglio SJ (2012). Microglial stimulation of glioblastoma invasion involves epidermal growth factor receptor (EGFR) and colony stimulating factor 1 receptor (CSF-1R) signaling. Mol Med.

[CR5] Cox LA (2006). Universality of J-shaped and U-shaped dose–response relations as emergent properties of stochastic transition systems. Dose Response.

[CR6] Dunn GP, Rinne ML, Wykosky J, Genovese G, Quayle SN, Dunn IF, Agarwalla PK, Chheda MG, Campos B, Wang A, Brennan C, Ligon KL, Furnari F, Cavenee WK, Depinho RA, Chin L, Hahn WC (2012). Emerging insights into the molecular and cellular basis of glioblastoma. Genes Dev.

[CR7] Franklin HR, Simonetti GP, Dubbelman AC, ten Bokkel Huinink WW, Taal BG, Wigbout G, Mandjes IA, Dalesio OB, Aaronson NK (1994). Toxicity grading systems. A comparison between the WHO scoring system and the Common Toxicity Criteria when used for nausea and vomiting. Ann Oncol.

[CR8] George D (2003). Targeting PDGF receptors in cancer–rationales and proof of concept clinical trials. Adv Exp Med Biol.

[CR9] Haberler C, Gelpi E, Marosi C, Rössler K, Birner P, Budka H, Hainfellner JA (2006). Immunohistochemical analysis of platelet-derived growth factor receptor-alpha, -beta, c-kit, c-abl, and arg proteins in glioblastoma: possible implications for patient selection for imatinib mesylate therapy. J Neurooncol.

[CR10] Hayes KE, Walk EL, Ammer AG, Kelley LC, Martin KH, Weed SA (2012). Ableson kinases negatively regulate invadopodia function and invasion in head and neck squamous cell carcinoma by inhibiting an HB-EGF autocrine loop. Oncogene.

[CR11] Hincal F, Gürbay A, Favier A (2003). Biphasic response of ciprofloxacin in human fibroblast cell cultures. Nonlinearity Biol Toxicol Med.

[CR12] Holdhoff M, Supko JG, Gallia GL, Hann CL, Bonekamp D, Ye X, Cao B, Olivi A, Grossman SA (2010). Intratumoral concentrations of imatinib after oral administration in patients with glioblastoma multiforme. J Neurooncol.

[CR13] Lennartsson J, Ronnstrand L (2012). Stem cell factor receptor/c-Kit: from basic science to clinical implications. Physiol Rev.

[CR14] Little SE, Popov S, Jury A, Bax DA, Doey L, Al-Sarraj S, Jurgensmeier JM, Jones C (2012). Receptor tyrosine kinase genes amplified in glioblastoma exhibit a mutual exclusivity in variable proportions reflective of individual tumor heterogeneity. Cancer Res.

[CR15] Macdonald DR, Cascino TL, Schold SC, Cairncross JG (1990). Response criteria for phase II studies of supratentorial malignant glioma. J Clin Oncol.

[CR16] Mader CC, Oser M, Magalhaes MA, Bravo-Cordero JJ, Condeelis J, Koleske AJ, Gil-Henn H (2011). An EGFR-Src-Arg-cortactin pathway mediates functional maturation of invadopodia and breast cancer cell invasion. Cancer Res.

[CR17] Minniti G, De Sanctis V, Muni R, Filippone F, Bozzao A, Valeriani M, Osti MF, De Paula U, Lanzetta G, Tombolini V, Maurizi ER (2008). Radiotherapy plus concomitant and adjuvant temozolomide for glioblastoma in elderly patients. J Neurooncol.

[CR18] Morris PG, Abrey LE (2010). Novel targeted agents for platelet-derived growth factor receptor and c-KIT in malignant gliomas. Target Oncol.

[CR19] Mouchemore KA, Pixley FJ (2012). CSF-1 signaling in macrophages: pleiotrophy through phosphotyrosine-based signaling pathways. Crit Rev Clin Lab Sci.

[CR20] Panjarian S, Iacob RE, Chen S, Engen JR, Smithgall TE (2013). Structure and dynamic regulation of Abl kinases. J Biol Chem.

[CR21] Raymond E, Brandes AA, Dittrich C, Fumoleau P, Coudert B, Clement PM, Frenay M, Rampling R, Stupp R, Kros JM, Heinrich MC, Gorlia T, Lacombe D, van den Bent MJ, European Organisation for Research and Treatment of Cancer Brain Tumor Group Study (2008). Phase II study of imatinib in patients with recurrent gliomas of various histologies: a European Organisation for Research and Treatment of Cancer Brain Tumor Group Study. J Clin Oncol.

[CR22] Razis E, Selviaridis P, Labropoulos S, Norris JL, Zhu MJ, Song DD, Kalebic T, Torrens M, Kalogera-Fountzila A, Karkavelas G, Karanastasi S, Fletcher JA, Fountzilas G (2009). Phase II study of neoadjuvant imatinib in glioblastoma: evaluation of clinical and molecular effects of the treatment. Clin Cancer Res.

[CR23] Soo GW, Law JH, Kan E, Tan SY, Lim WY, Chay G, Bukhari NI, Segarra I (2010). Differential effects of ketoconazole and primaquine on the pharmacokinetics and tissue distribution of imatinib in mice. Anticancer Drugs.

[CR24] Stupp R, Hegi ME, Mason WP, van den Bent MJ, Taphoorn MJ, Janzer RC, Ludwin SK, Allgeier A, Fisher B, Belanger K, Hau P, Brandes AA, Gijtenbeek J, Marosi C, Vecht CJ, Mokhtari K, Wesseling P, Villa S, Eisenhauer E, Gorlia T, Weller M, Lacombe D, Cairncross JG, Mirimanoff RO, European Organisation for Research and Treatment of Cancer Brain Tumour and Radiation Oncology Groups (2009). Effects of radiotherapy with concomitant and adjuvant temozolomide versus radiotherapy alone on survival in glioblastoma in a randomised phase III study: 5-year analysis of the EORTC-NCIC trial. Lancet Oncol.

[CR25] Szerlip NJ, Pedraza A, Chakravarty D, Azim M, McGuire J, Fang Y, Ozawa T, Holland EC, Huse JT, Jhanwar S, Leversha MA, Mikkelsen T, Brennan CW (2012). Intratumoral heterogeneity of receptor tyrosine kinases EGFR and PDGFRA amplification in glioblastoma defines subpopulations with distinct growth factor response. Proc Natl Acad Sci U S A.

[CR26] Tan SY, Kan E, Lim WY, Chay G, Law JH, Soo GW, Bukhari NI, Segarra I (2011). Metronidazole leads to enhanced uptake of imatinib in brain, liver and kidney without affecting its plasma pharmacokinetics in mice. J Pharm Pharmacol.

[CR27] Taphoorn MJ, Bottomley A (2005). Health-related quality of life and symptom research in glioblastoma multiforme patients. Expert Rev Pharmacoecon Outcomes Res.

[CR28] Trotti A (2003). CTCAE v3.0: development of a comprehensive grading system for the adverse effects of cancer treatment. Semin Radiat Oncol.

[CR29] Verhaak RG, Hoadley KA, Purdom E, Wang V, Qi Y, Wilkerson MD, Miller CR, Ding L, Golub T, Mesirov JP, Alexe G, Lawrence M, O’Kelly M, Tamayo P, Weir BA, Gabriel S, Winckler W, Gupta S, Jakkula L, Feiler HS, Hodgson JG, James CD, Sarkaria JN, Brennan C, Kahn A, Spellman PT, Wilson RK, Speed TP, Gray JW, Meyerson M, Getz G, Perou CM, Hayes DN, Cancer Genome Atlas Research Network (2010). Integrated genomic analysis identifies clinically relevant subtypes of glioblastoma characterized by abnormalities in PDGFRA, IDH1, EGFR, and NF1. Cancer Cell.

[CR30] Waller CF (2010). Imatinib mesylate. Recent results. Cancer Res.

[CR31] Wen PY, Yung WK, Lamborn KR, Dahia PL, Wang Y, Peng B, Abrey LE, Raizer J, Cloughesy TF, Fink K, Gilbert M, Chang S, Junck L, Schiff D, Lieberman F, Fine HA, Mehta M, Robins HI, DeAngelis LM, Groves MD, Puduvalli VK, Levin V, Conrad C, Maher EA, Aldape K, Hayes M, Letvak L, Egorin MJ, Capdeville R, Kaplan R, Murgo AJ, Stiles C, Prados MD (2006). Phase I/II study of imatinib mesylate for recurrent malignant gliomas: North American Brain Tumor Consortium Study 99-08. Clin Cancer Res.

[CR32] Wöhrer A, Waldhör T, Heinzl H, Hackl M, Feichtinger J, Gruber-Mösenbacher U, Kiefer A, Maier H, Motz R, Reiner-Concin A, Richling B, Idriceanu C, Scarpatetti M, Sedivy R, Bankl HC, Stiglbauer W, Preusser M, Rössler K, Hainfellner JA (2009). The Austrian Brain Tumour Registry: a cooperative way to establish a population-based brain tumour registry. J Neurooncol.

